# Fabrication of Sericin/Agrose Gel Loaded Lysozyme and Its Potential in Wound Dressing Application

**DOI:** 10.3390/nano8040235

**Published:** 2018-04-13

**Authors:** Meirong Yang, Yejing Wang, Gang Tao, Rui Cai, Peng Wang, Liying Liu, Lisha Ai, Hua Zuo, Ping Zhao, Ahmad Umar, Chuanbin Mao, Huawei He

**Affiliations:** 1 State Key Laboratory of Silkworm Genome Biology, Southwest University, Beibei, Chongqing 400715, China; yangmeirong@email.swu.edu.cn (M.Y.); taogang@email.swu.edu.cn (G.T.); l3341345@email.swu.edu.cn (L.L.); als123@email.swu.edu.cn (L.A.); zhaop@swu.edu.cn (P.Z.); 2 College of Biotechnology, Southwest University, Beibei, Chongqing 400715, China; cairui0330@email.swu.edu.cn (R.C.); modelsums@email.swu.edu.cn (P.W.); 3 College of Pharmaceutical Sciences, Southwest University, Beibei, Chongqing 400715, China; zuohua@swu.edu.cn; 4 Chongqing Engineering and Technology Research Center for Novel Silk Materials, Southwest University, Beibei, Chongqing 400715, China; 5 Department of Chemistry, College of Science and Arts and Promising Centre for Sensors and Electronics Devices (PCSED), Najran University, P.O. Box 1988, Najran 11001, Saudi Arabia; umahmad@nu.edu.sa; 6 Department of Chemistry & Biochemistry, Stephenson Life Science Research Center, University of Oklahoma, 101 Stephenson Parkway, Norman, OK 73019, USA; cbmao@ou.edu; 7 School of Materials Science and Engineering, Zhejiang University, Hangzhou 310027, China

**Keywords:** silk sericin, agarose, lysozyme, composite gel, wound dressing

## Abstract

Sericin is a biomaterial resource for its significant biodegradability, biocompatibility, hydrophilicity, and reactivity. Designing a material with superabsorbent, antiseptic, and non-cytotoxic wound dressing properties is advantageous to reduce wound infection and promote wound healing. Herein, we propose an environment-friendly strategy to obtain an interpenetrating polymer network gel through blending sericin and agarose and freeze-drying. The physicochemical characterizations of the sericin/agarose gel including morphology, porosity, swelling behavior, crystallinity, secondary structure, and thermal property were well characterized. Subsequently, the lysozyme loaded sericin/agarose composite gel was successfully prepared by the solution impregnation method. To evaluate the potential of the lysozyme loaded sericin/agarose gel in wound dressing application, we analyzed the lysozyme loading and release, antimicrobial activity, and cytocompatibility of the resulting gel. The results showed the lysozyme loaded composite gel had high porosity, excellent water absorption property, and good antimicrobial activities against *Escherichia coli* and *Staphylococcus aureus.* Also, the lysozyme loaded gel showed excellent cytocompatibility on NIH3T3 and HEK293 cells. So, the lysozyme loaded sericin/agarose gel is a potential alternative biomaterial for wound dressing.

## 1. Introduction

Non-healing skin wounds exposed to bacterial infections are biologically characterized by lengthening inflammation, interfering re-epithelialization, disturbing collagen production, and finally delaying wound healing [1]. In wound care, wound dressing is an important biomedical material used to protect the wound from infection and facilitate wound healing [2]. Accompanied by the growing number of chronic diseases, the wound dressing market is evolving rapidly in the present healthcare system worldwide [3]. The ideal wound dressing should absorb wound exudate in a manner, allow gas exchange and maintain necessary moisture at wound interface without cytotoxicity and allergenic response [4]. Besides, it can promote wound healing by creating a suitable microenvironment to prevent bacterial infection and promote cell adhesion and proper proliferation [5]. Among all wound dressing materials, hydrogel is an attractive alternative in traditional therapeutic approaches for its multifunctional abilities such as hydrophilicity, swelling, drug delivery, and in situ gelling capacity [6].

Sericin (SS) is one of the major protein components of silk, which is discarded as a waste during the degumming process in the textile industry [7]. Sericin is a natural protein, exhibiting immense potential in the field of biomaterial owing to its biodegradability, easy availability, and hydrophilicity [8]. Sericin has numerous biological activities such as anti-oxidation, anti-bacterium, and anti-coagulation, promoting cell growth and differentiation [9]. However, sericin is physically fragile and highly soluble due to its amorphous nature [10], which is unsuitable for biomedical applications. Hence, in order to obtain desired material with improved properties for regenerative medicine application, sericin is mostly designed to copolymerize, crosslink, or blend with other polymers as it has polar side chains with diverse functional groups, such as amine, hydroxyl, and carboxyl groups [11,12,13,14,15]. Agarose (AR) is a transparent, neutrally charged, and thermo-reversible natural polysaccharide [16]. Agarose is used extensively in vitro cartilage tissue engineering as it provides a superior foundation for chondrogenesis and higher glycosaminoglycan deposition to produce constructs with functional properties approaching those of native articular cartilage [17,18]. Additionally, agarose gel is considered as a biological scaffold material for the central nervous system repair and regeneration due to its excellent mechanical properties which can well match the growth and control of nerve axis, the porous structure which is conducive to nutrient delivery, and the implant which does not cause adverse reactions [19]. However, agarose shows low cell adhesiveness and cell proliferation activity in vivo [20]. Therefore, blending agarose with other polymers such as chitosan and gelatin to overcome the valid drawbacks has escalated in recent years [21,22]. Consequently, the present study brought together the innate advantages of sericin and agarose to fabricate a blended hydrogel for prospective application in a wound dressing.

Nevertheless, the hydrogel is limited as a wound dressing material because it may paradoxically provide a preferred environment for infectious bacteria. To prevent bacterial infection on both skin wound and dressing material, antibiotics such as penicillin and methicillin have been widely used. However, the widespread and indiscriminate use of antibiotics now constitutes a major health concern worldwide due to the emergence of numerous resistant pathogens [23]. Therefore, tremendous attention has been paid to the discovery and development of alternative novel antibiotics, particularly with new modes of action to overcome the resistance. Lysozyme is a natural antibacterial agent that has been isolated from the cells and secretions of virtually all the living organisms [24]. Lysozyme plays the role of the anti-microbial agent through catalyzing the hydrolysis of β-1,4 glycosidic bonds between N-acetylmuramic acid and N-acetylglucosamine in peptidoglycans of the bacterial cell wall [25]. It is commercially available at low cost, and classified as GRAS grade by the Food and Drug Administration (FDA, US) and as a food additive by the European Union (E 1105) [26]. Lysozyme has been extensively applied as antibacterial agents in wound dressing and protein separation [27]. Accordingly, the development of lysozyme-based antimicrobial material is significantly important toward an environmentally benign antimicrobial field.

We herein developed an improved lysozyme (LZM) loaded sericin/agarose (SS/AR) gel (SS/AR/LZM). Scanning Electronic Microscopy (SEM), Attenuated Total Reflection Fourier Transform Infrared Spectroscopy (ATR-FTIR), X-ray Diffraction (XRD), Thermogravimetric Analysis (TGA), and swelling behavior test were performed to characterize the physicochemical properties of SS/AR gel. We successfully fabricated lysozyme loaded SS/AR composite biomaterials by solution impregnation method. We investigated the lysozyme loading and release, the antimicrobial activity of SS/AR/LZM gel against typical Gram negative/positive bacteria *Escherichia coli* (*E. coli*) and *Staphyloccus aureus* (*S. aureus*). In addition, the cytotoxicity of the lysozyme loaded SS/AR gel was evaluated on NIH3T3 and HEK293 cells. The results suggested that the SS/AR/LZM gel with antimicrobial activity and cytocompatibility has a great potential in wound dressing application.

## 2. Materials and Methods

### 2.1. Materials

*Bombyx mori* cocoons were provided by the State Key Laboratory of Silkworm Genome Biology, Southwest University (China, 400716). Lysozyme (20,000 U/mg) was obtained from Sangon Biotech Co. Ltd. (Shanghai, China). Agarose G-10 was purchased from Biowest (Nuaillé, France). Cell counting kit-8 (CCK-8) was from Beyotime (Beijing, China). LIVE/DEAD cell viability kit was from Thermo Fisher Scientific (Waltham, MA, USA). NIH3T3 (mouse embryonic fibroblast) and HEK293 (human embryonic kidney) cell lines were obtained from China Infrastructure of Cell Line Resources. Chemicals for cell culture such as Dulbecco’s modified Eagle’s medium (DMEM), Fetal Bovine Serum (FBS), Trypsin-EDTA and Penicillin/Streptomycin were from Gibco BRL (Gaithersburg, MD, USA). Ultrapure water was the product of Milli-Q Plus system from Millipore (Billerica, MA, USA). All other chemicals utilized were of analytical grade.

### 2.2. Fabrication of SS/AR Gel

Sericin was extracted from *Bombyx mori* cocoons as previous reports [28,29]. Briefly, silkworm cocoons were cut into pieces and autoclaved at 121 °C for 30 min to obtain sericin solution. Subsequently, sericin solution was freeze-drying to become sericin powder, and then dissolved in hot water. Sericin and agarose solution (2%, *w*/*v*) were mixed gently, and allowed to gel after casting into 24-well cell culture plates. These stable hydrogels were then frozen at −80 °C for 12 h followed by lyophilization for 24 h to become gels. According to the volume ratios of sericin and agarose solution, the corresponding SS/AR gels were termed as S100A0, S75A25, S50A50, S25A75, and S0A100, respectively.

### 2.3. Characterization of SS/AR Gel

SEM observation was performed on JSM-5610LV (Tokyo, Japan) with working voltage of 25 kV to examine the surface morphologies of the gel. The porosities of SS/AR gels were calculated according to the liquid displacement method [30]. Briefly, SS/AR gel was immersed into water (V1) in a graduated cylinder, the total volume including water and SS/AR gel was recorded as V2. The SS/AR gel was then removed from the cylinder and the residual water volume was recorded as V3. The porosity (*p*) of SS/AR gel was calculated using the following equation:

*p* = (V_1_ − V_3_)/(V_2_ − V_3_) × 100%. (1)

ATR-FTIR spectra of sericin and SS/AR gel were determined in the wavenumber range of 650–4000 cm^−1^ at a resolution of 4 cm^−1^ on a Nicolet iz10 Infrared spectrophotometer from Thermo Fisher Scientific (Waltham, MA, USA). XRD of sericin and SS/AR gel were carried out by X’Pert powder X-ray diffraction system (PANalytical, Almelo, OV, Netherland) within a 2θ range of 10°–70°. The thermal behaviors of sericin and SS/AR gel were analyzed by a thermogravimetric analyzer TGA-Q50 (TA instruments, New Castle, DE, USA) under a nitrogen flow of 20 mL/min [31]. The specimens were heated from room temperature to 600 °C, at a heating rate of 10 °C/min.

### 2.4. Swelling Behavior

The swelling ability of the SS/AR gel was analyzed using a conventional gravimetric method [32]. Briefly, a pre-weighed dry gel (Wd) was immersed into water at 37 °C for 30 min to achieve equilibrium. The swollen weight of gel was recorded as Ws at specific time intervals. The experiment was repeated for three times under the same conditions. Swelling ratios (S) were determined as the following equation:

S = (Ws − Wd)/Wd × 100%.(2)

### 2.5. Preparation of SS/AR/LZM Gel

To prepare the lysozyme loaded SS/AR gel, S50A50 was cut into a circular piece with a diameter of 1.5 cm and then immersed into lysozyme solution (20–75 mg/mL) for 16 h. Subsequently, the lysozyme loaded SS/AR gel was removed from lysozyme solution and freeze-dried. According to the lysozyme concentration, the resulting SS/AR/LZM gels were termed as S50A50L20, S50A50L50 and S50A50L75, respectively.

### 2.6. The Loading and Release of Lysozyme

Lysozyme has a specific absorption peak at 280 nm [33], which could be used to measure the loaded and released lysozyme concentration. Ultraviolet visible spectrophotometer was employed to analyze the loading and release of lysozyme. The loaded lysozyme content was determined by the difference of lysozyme concentration before and after the treatment. The circular SS/AR/LZM gel with a diameter of 1.5 cm was dispensed into a centrifuge tube containing 4 mL of 0.01 M PBS (pH 7.4) buffer at 37 °C. At special time points, an aliquot (1 mL) PBS buffer was collected to measure the absorbance at 280 nm to determine the released lysozyme contents. Then the gel was transferred into 4 mL fresh PBS solution for the next measurement. The cumulative release rate was determined according to the ratio of the released and loaded lysozyme. Various lysozyme concentrations (0.1–0.6 mg/mL) were prepared for the calibration curve. All experiments were performed in triplicate.

### 2.7. In Vitro Antibacterial Assay

The antibacterial activity was evaluated according to the previous procedures with a slight modification [34,35]. SS/AR and SS/AR/LZM gels were cut pieces with 1 mm in thick and 1.5 cm in diameter, subsequently sterilized with UV radiation for 30 min. *E. coli* and *S. aureus* were grown in Luria–Bertani (LB) media at 37 °C with continuous shaking until the optical density at 600 nm (OD_600_) reached about 1.5. Bacteria (500 μL) were harvested by centrifugation at 1000 rpm for 5 min followed by washing with 0.01 M phosphate buffer saline (PBS, pH 7.4). Subsequently, bacteria were re-suspended and diluted with PBS buffer. Next, 50 µL of the diluted bacterial suspension was cultured at 37 °C for 2 h in the presence of SS/AR gel or SS/AR/LZM gel. Aliquots (1 µL) of the mixture were diluted (1:10,000) in PBS, and then manually spread on LB agar plates. After 16 h incubation at 37 °C, the units of colony formation in each agar plate were counted to check the antibacterial ability of the gels. Each independent experiment was performed in triplicate.

### 2.8. Cytocompatibility Assay

NIH3T3 and HEK293 cells were cultured in high glucose DMEM supplemented with 10% FBS and 1% penicillin/streptomycin in a humidified atmosphere of 95% and 5% CO_2_ at 37 °C. To check the cell viability, NIH3T3 and HEK293 cells (100 μL) were loaded at the density of 1 × 10^4^ cells/well in 96-well plates and incubated 12 h at 37 °C. SS/AR or SS/AR/LZM gel was sterilized by ultraviolet radiation overnight and then added to the cell plates. Non-treated cells were used as a control.

After treated with the gels for 12 h, 24 h and 36 h, CCK-8 assay was used to assess cell viability according to the manufacturer’s instructions. CCK-8 solution (10 µL) was added into each well and then incubated at 37 °C for 1.5 h. The optical density (OD) of each well was measured at 450 nm on a microplate reader TECAN (Mannedorf, Switzerland). The cell viability is defined as the percentage of OD value of the treated and control wells. For each experiment, at least three samples were evaluated. The morphologies of NIH3T3 and HEK293 cells after incubation for 24 h in the absence and presence of SS/AR or SS/AR/LZM gel were observed on a fluorescence microscope EVOS FL Auto Cell Imaging System (Life, Bothell, WA, USA).

Additionally, LIVE/DEAD staining assay was carried out to further assess the effects of the gels on the cells viability. NIH3T3 and HEK293 cells were cultured and incubated at 37 °C as described above. The cells after treated with SS/AR or SS/AR/LZM gel for 24 h were incubated with staining solution at 37 °C for 15 min. Then the images were collected on EVOS FL Auto Cell Imaging System. For each sample, the experiment was done in triplicate.

## 3. Results and Discussion

### 3.1. Preparation of SS/AR/LZM Gel

**Figure 1:**
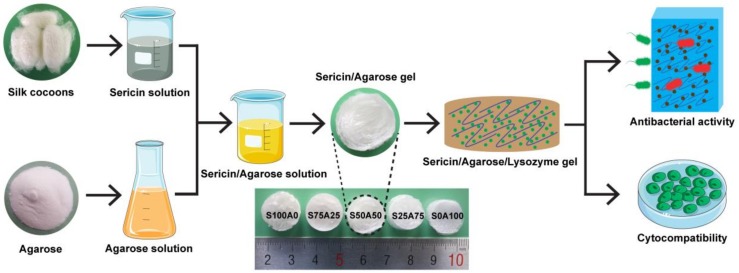
Schematic illustration of the fabrication of sericin (SS)/agarose (AR)/lysozyme (LZM) gel.

In this study, we prepared the SS/AR/LZM gel with good antibacterial activity and cytocompatibility, as illustrated in Figure 1. Sericin and agarose solution were mixed and then freeze-dried to become a porous gel. Thereafter, lysozyme, a natural antimicrobial agent, was loaded into the SS/AR gel. As sericin is negatively charged, agarose is neutral, whereas lysozyme is positively charged, thus the adsorption of lysozyme into the SS/AR gel may be attributed to the electrostatic interactions between the opposite charges of sericin and lysozyme [36]. Also, the physical adsorption caused by free diffusion could promote the adsorption of lysozyme. In addition, lysozyme has carboxyl group, amino groups and four disulfide bonds [37], and sericin has hydroxyl, carboxyl, and amino groups [38]. The special hydrophilic/hydrophobic interactions between lysozyme and sericin are also able to enhance the adsorption of lysozyme. The resulting SS/AR/LZM gel with interconnected porous structures, high swelling ability, good antibacterial activity and cytocompatibility may be a prospective alternative for wound dressing.

### 3.2. Morphology of SS/AR Gel

**Figure 2:**
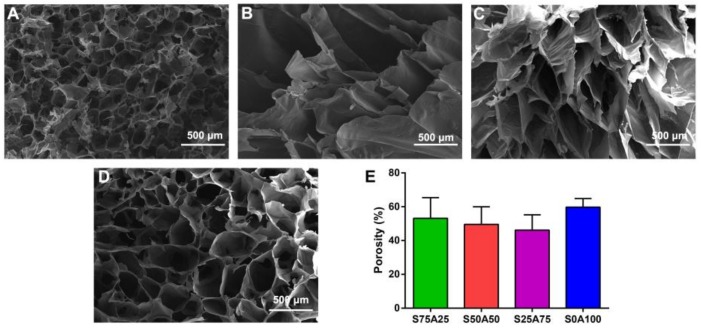
The porous microstructures of S75A25 (**A**), S50A50 (**B**), S25A75 (**C**), and S0A100 (**D**) gels. The porosity of gels with different ratios of sericin and agarose (**E**).

Porous materials provide space for cell growth and proliferation, and the microenvironment for the retention and release of bioactive molecules [39]. Furthermore, the porous structure affects the supply of nutrients and oxygen, and the removal of wastes [40], which is of utmost importance to wound dressing. In this study, the prepared gels had macro-porous “open-cell” structures (Figure 2A–D). The porosity of S75A25, S50A50, S25A75, and S0A100 gels were 53.17%, 49.54%, 46.17% and 59.75%, respectively (Figure 2E). Compared to other gels, S50A50 and S25A75 gels had significantly bigger pore sizes. This may be the fact that S50A50 and S25A75 gels could adsorb more water. After lyophilization, the space water occupied resulted in the formation of pores. Consequently, the pore size and porosity of gels were dependent on the ratio of sericin and agarose solution.

### 3.3. Characterization of SS/AR Gel

**Figure 3:**
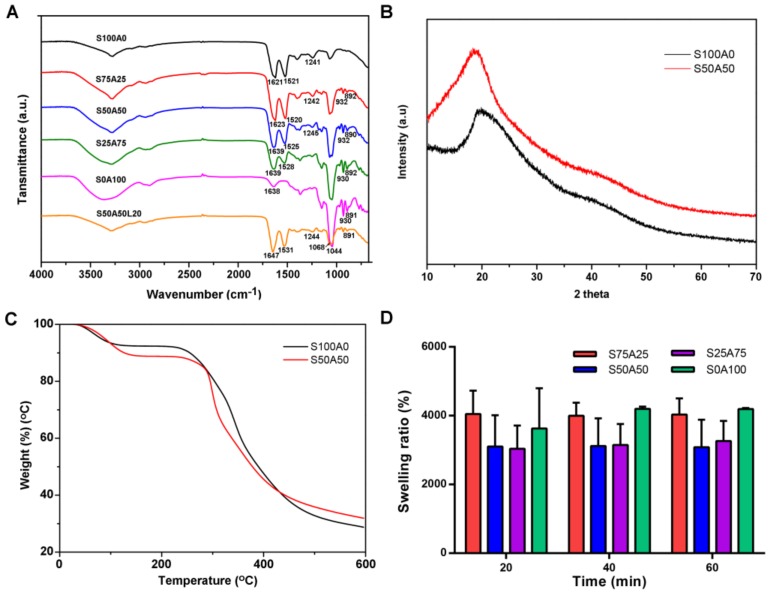
Characterizations of SS/AR gels. (**A**) Attenuated Total Reflection Fourier Transform Infrared Spectroscopy (ATR-FTIR); (**B**) X-Ray Diffraction (XRD); (**C**) Thermogravimetric Analysis (TGA); and (**D**) swelling ratio.

ATR-FTIR was employed to analyze the chemical interactions between sericin and agarose as ATR technique can probe to only a shallow depth and thus emphasize any surface coatings [41]. Sericin has a typical spectrum with distinctive peaks at 1600–1700 cm^−1^ (amide I, C=O stretching vibration), 1480–1575 cm^−1^ (amide II, N–H bending vibration), and 1229–1301 cm^−1^ (amide III) [42]. As shown in Figure 3A, sericin gel had characteristic peaks at 1621 cm^−1^, 1521 cm^−1^, and 1241 cm^−1^, corresponding to amide I, amide II, and amide III, respectively. Pure agarose exhibited its characteristic peaks at 1068 cm^−1^ (C–O, axial deformation), 930 cm^−1^ (3, 6-anhydro-galactose), and 891 cm^−1^ (C–H, angular deformation of β anomeric carbon), the result was consistent with the previous study [43]. In the blended gels, the characteristic peaks of both agarose and sericin were recorded, which confirmed the presence of both components. Some slight shifts in amide I and amide II peaks, and the differences in the intensity of peaks were observed in case of the composite gels, which indicated that the backbone structures of sericin and agarose did not change. Lysozyme has characteristic peaks at 3295 cm^−1^ (NAH stretching of the free amino groups), and 2961 cm^−1^ (CAH stretching) as well7 as amide I (1600–1700 cm^−1^), amide II (1500–1600 cm^−1^) and amide III (1230–1320 cm^−1^) [44]. Few peaks were found to overlap with the peaks of sericin.

The crystalline structure of the composite was analyzed by XRD. Silk protein has main diffraction peaks of Silk I (2θ = 12.2° and 28.2°), and Silk II (2θ = 18.9° and 20.7°) [45]. Similar XRD patterns of sericin with peaks at 2θ = 19.2° and 21.15° have been reported [46,47,48,49]. Sericin and SS/AR gel exhibited obvious diffraction peaks at 19.08° and 19.56°, respectively (Figure 3B), which indicated the existence of sericin in the SS/AR gel. The difference of 2θ between sericin and SS/AR gel reflect the conversion of the random coil to β-sheet structure for the presence of the intermolecular hydrogen bond in sericin [50].

The thermal stability of sericin and SS/AR gel were examined by TGA. Sericin and SS/AR gel underwent three stages of thermal degradation including dehydration, deploymerization, and decomposition (Figure 3C). The first stage was from room temperature to around 110 °C, where the mass loss revealed the removal of adsorbed water molecules in sericin and SS/AR gel. The second major decomposition was attributed to the degradation of sericin and agarose occurred in the temperature range of 120–410 °C. At this stage, the mass loss of SS/AR gel was faster than that of sericin, indicating that sericin could improve the thermal stability of the composite gels and delay the thermal degradation process. At the last stage, the mass loss occurred from 420 °C to 600 °C, which was associated with the breakdown of sericin and agarose.

### 3.4. Swelling Behavior

Swelling ratio is a vital property to illustrate the uptake of liquids in wound dressing. The swelling ratio of various SS/AR gels were shown in Figure 3D. The results showed that all samples exhibited good swelling behavior. After 60 min, S75A25 and S0A100 gels had the swelling ratios of 3628–4196%, whereas S50A50 and S25A75 had the swelling ratios of 3040–3262%. The swelling ratios of S75A25 and S0A100 gels were higher than those of S50A50 and S25A75 as they had smaller pore size and higher porosities. The swelling of the gels had two stages, including the growing period and equilibrium period. In the initial 20 min, the swelling ratios of all samples increased quickly, indicating the excellent hydrophilicity of the composite gels. Thereafter, all gels quickly reached the swelling equilibrium.

The SS/AR gels with various ratios had a honeycomb structure, high porosity as well as excellent swelling capacity. In this study, we purposed to develop an alternative wound dressing through bringing together the innate advantages of sericin and agarose. High content of sericin will reduce the mechanical property of the gel, and high content of agarose may affect the cell adhesion and proliferation. Hence, we suggested the SS/AR gel with a ratio of 50:50 (S50A50) had moderate mechanical property and cytocompatibility, which may be suitable for wound dressing. The S50A50 gel was chosen for the further experiments.

### 3.5. Lysozyme Release

**Figure 4:**
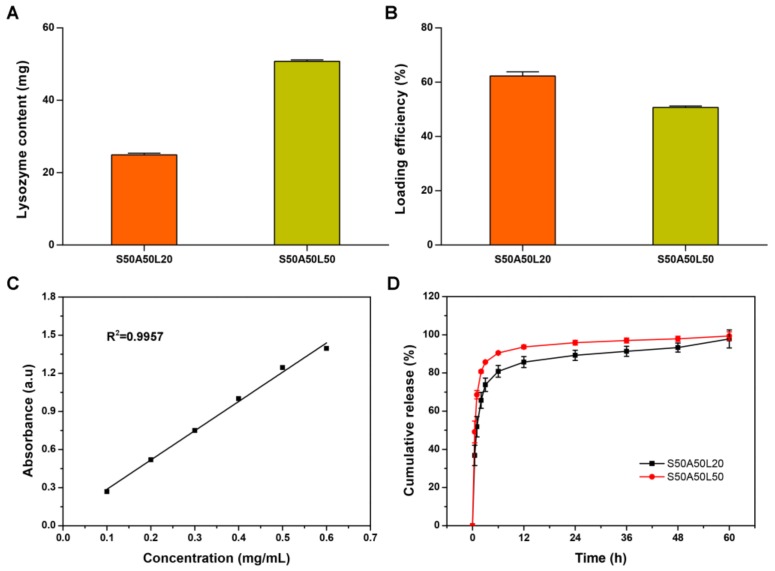
The loading and release of lysozyme. (**A**) Lysozyme contents loaded on SS/AR gel; (**B**) loading efficiency; (**C**) standard curves of UV intensity and lysozyme concentration; and (**D**) the cumulative release of lysozyme.

To avoid frequent replacement of the dressing and reduce the risk of overexposing wound to bacteria, a wound dressing should have a controllable drug release ability [51,52]. The loaded lysozyme contents of S50A50L20 and S50A50L50 gels were 24.94 mg and 50.78 mg, respectively (Figure 4A). And the loading efficiency of lysozyme was 62% and 51% for S50A50L20 and S50A50L50 gels, respectively (Figure 4B). Increasing lysozyme solution concentration could increase the loaded lysozyme contents, however, reduce its loading efficiency. To assess lysozyme release, we analyzed the correlation between lysozyme concentration and UV absorption by the standard curve. The results showed that UV absorption was tightly correlated with lysozyme concentration (Figure 4C). Obviously, lysozyme could be released from both S50A50L20 and S50A50L50 gels. Lysozyme release could be divided into burst stage and steady stage (Figure 4D). During the initial burst phase, the release rate was relatively high. This effect is associated with the diffusion of water molecules and desorption of lysozyme close to the surface of the gel [53]. At the steady phase, lysozyme was gradually released from SS/AR/LZM gels up to 60 h. The cumulative release of S50A50L20 and S50A50L50 gels reached 74% and 86% at 3 h, respectively. After 60 h, the cumulative release reached 98% and 99%, indicating lysozyme was almost entirely released from the composite gels. The results suggested that the SS/AR/LZM gel had a sustainable lysozyme releasing ability, which is required for a wound dressing.

### 3.6. Antibacterial Activity

**Figure 5:**
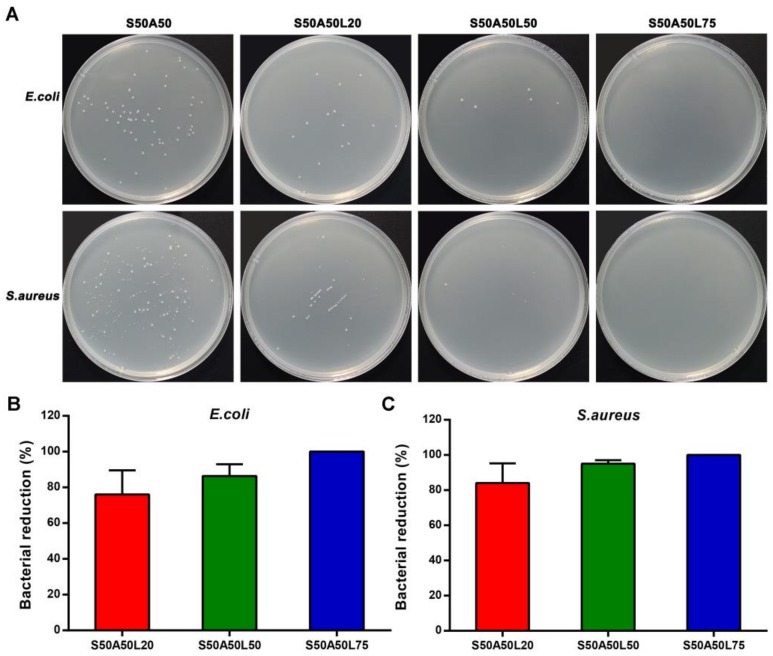
Antibacterial activities of SS/AR/LZM gels against *E. coli* and *S. aureus*. (**A**) Total bacterial colonies counting; (**B**, **C**) Bacterial colonies reduction rate.

The antibacterial activity of the composite gels toward *E. coli* and *S. aureus* were shown in Figure 5A. Compared to the control, the total colonies number in the presence of SS/AR/LZM gels significantly decreased, indicating the SS/AR/LZM gel had good antibacterial activity. The S50A50L20 and S50A50L50 gels exhibited the bacteria reduction rates of 76%/87% and 84%/95% toward *E. coli*, *S. aureus*, respectively. The S50A50L75 gel completely inhibited the growth of the bacteria both for *E. coli* and *S. aureus*, suggesting increasing lysozyme solution concentration can improve the bactericidal ability of the composite gel as it increased the loaded and released lysozyme contents.

Lysozyme is a natural antibacterial agent. Its antibacterial activity is mild compared with inorganic and organic antibacterial agents. To improve the antibacterial effect of lysozyme, some strategies such as physical and chemical modifications or synergistic action with other substances have been developed in recent years [26,54,55,56]. In this study, the SS/AR/LZM gels for antibacterial test had a dimension of 1 mm in thickness and 1.5 cm in diameter. The size and thickness determined the low loading content of lysozyme. In addition, the bacterial suspension volume affected the release efficiency of lysozyme from the gels. Therefore, SS/AR/LZM gels exhibited expected antibacterial effect; however, the loading content and the release efficiency of lysozyme determined its antibacterial activity.

Various antimicrobial materials have been developed for wound dressing application, such as metals/metal oxides [57], antibiotics [58], and peptides [2]. These materials are associated with a big concern about adverse effects on ecosystems. For instance, silver nanoparticle, one of the most classical and important materials, exert an adverse effect on environmental safety during its preparation and application. Also, the widespread and indiscriminate use of antibiotics response to the emergence of numerous resistant pathogens. The cost of the synthetic antibacterial peptide is very high. Here, the developed SS/AR/LZM material has good cytocompatibility and without toxic side effects and drug resistance. The main advantage of the lysozyme-based material is significantly safe to human and environment, which has exciting and expected potentials in biomedical materials such as wound dressing.

### 3.7. Cytocompatibility Assay

**Figure 6:**
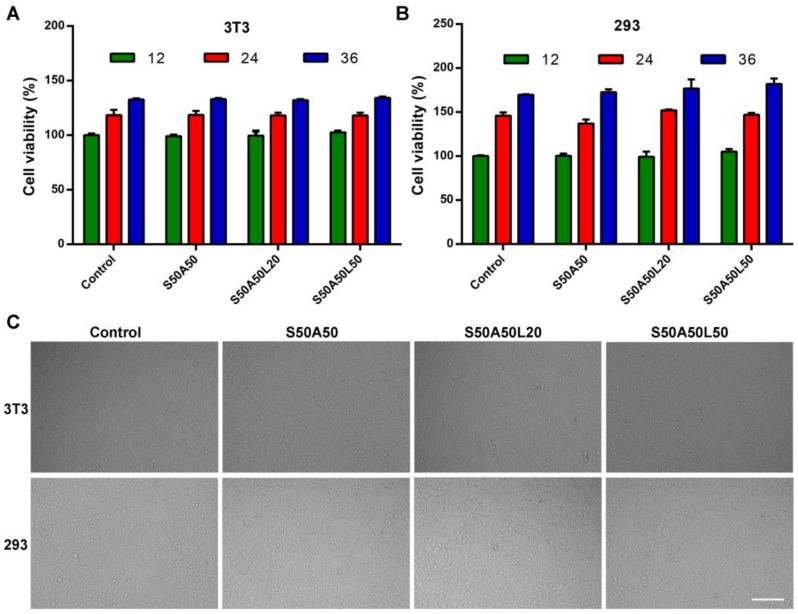
CCK-8 assay of the SS/AR/LZM gels. Cells viability of NIH3T3 (**A**) and HEK293 (**B**) in the presence of SS/AR gel or SS/AR/LZM gel, respectively. Microscopic analysis of NIH3T3 and HEK293 cells; ((**C**), scale bar, 200 μm).

To evaluate the cytocompatibility of SS/AR and SS/AR/LZM gels, we tested the effects of SS/AR and SS/AR/LZM gels on the viability of NIH3T3 and HEK293 cells. In the CCK-8 test, a soluble formazan dye with the maximum absorbance at 450 nm produced as metabolically active cells react with a tetrazolium salt in the CCK-8 reagent. And, the higher optical density (OD) value indicates better cell viability and more live cells [59]. As shown in Figure 6A, B, after 12 h, there were no significant differences in the cells viabilities among SS/AR, SS/AR/LZM gels and the control. After 24 h, the cells viabilities for SS/AR, SS/AR/LZM, and control groups increased quickly. After 36 h, the cells in all groups exhibited significantly higher viabilities than those at 24 h. The results suggested the SS/AR/LZM gel had good cytocompatibility on NIH3T3 and HEK293 cells. This may be due to the fact that active sericin promotes cell growth for its cytoprotective and mitogenic abilities [9]. Furthermore, the morphologies of NIH3T3 and HEK293 cells were observed on an optical microscope after 24 h in the absence and presence of SS/AR and SS/AR/LZM gels. The results showed that morphologies of the cells were nearly identical to that of the control (Figure 6C), indicating SS/AR and SS/AR/LZM gels were not toxic on NIH3T3 and HEK293 cells.

**Figure 7:**
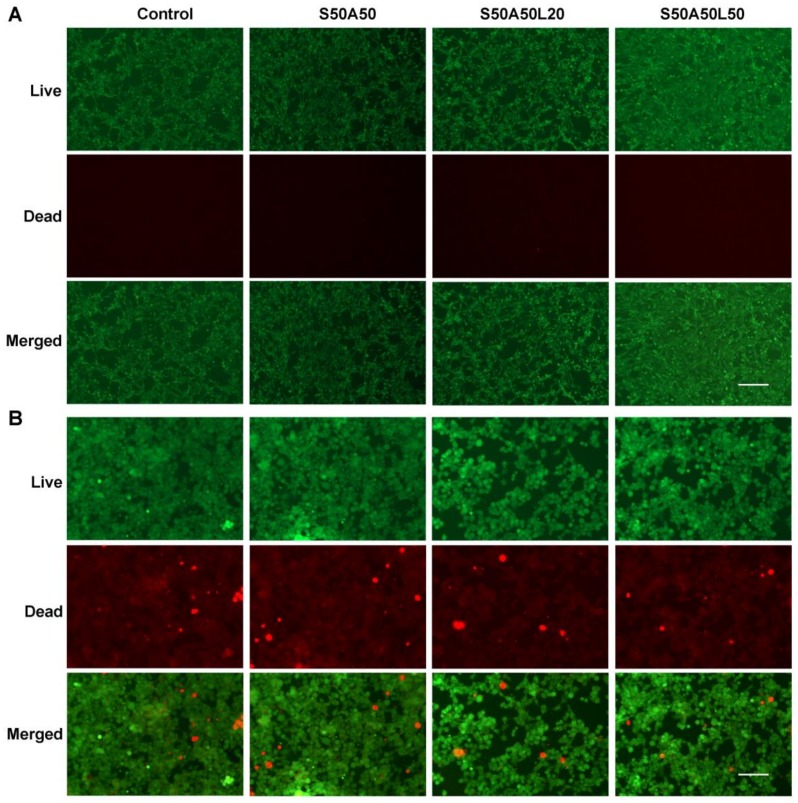
The LIVE/DEAD staining assay. NIH3T3 ((**A**), scale bar, 200 μm) and HEK293 ((**B**), scale bar, 100 μm) cells.

After staining with the LIVE/DEAD fluorescent reagent, the alive cells were stained green while the apoptotic cells were stained red. The result showed most of the cells were green and only a few of cells were red (Figure 7), indicating that the SS/AR and SS/AR/LZM gels had good cytocompatibility on NIH3T3 and HEK293 cells. The fluorescence images of the LIVE/DEAD staining assay were in accordance with the results of CCK-8 assay.

## 4. Conclusions

In summary, the SS/AR composite gel was successfully prepared by blending and freeze-drying. The SS/AR gel had highly porous and inter-connected structure, and good swelling behavior. The lysozyme loaded SS/AR gel had a sustainable lysozyme releasing ability and good antimicrobial activities against *E. coli* and *S. aureus* as well as excellent cytocompatibility on NIH3T3 and HEK293 cells. The SS/AR/LZM gel is expected to develop as an alternative for wound dressing.
